# Submental Artery Island Flap in Reconstruction of Hard Palate after wide Surgical Resection of Verruccous Carcinoma, Two Case Reports

**Published:** 2013-06

**Authors:** Amin Rahpeyma, Saeedeh Khajehahmadi, Mohammadreza Nakhaei

**Affiliations:** 1 *Oral and Maxillofacial Diseases Research Center, School of Dentistry, Mashhad University of Medical Sciences, Mashhad, Iran. *; 2 *Dental Research Center, School of Dentistry, Mashhad University of Medical Sciences, Mashhad, Iran*.

**Keywords:** Facial artery, Hemimaxillectomy, Surgical flaps

## Abstract

**Introduction:**

Reconstruction of intraoral soft tissue defects is important in restoring function and esthetic. In large defects, there will be demand for regional pedicle flaps or free flaps. Hard palate separates nasal and oral cavities. Due to the small surface area between flap and remaining palate after surgical resections, optimal blood supply of the flaps for hard palate reconstructions are needed.

**Case Report:**

This article demonstrates immediate reconstruction of two edentulous hemimaxillectomy patients with submental artery Island flap and brief review of this flap discussed.

**Conclusion:**

Submental Artery Island flap is an effective and reliable method for intraoral reconstruction of large soft tissue defects of oral cavity. Donor site morbidity is low and remaining scar is inconspicuous. Head and neck surgeons familiar with facial artery and its branching pattern make this flap an appropriate choice for clinical practice.

## Introduction

Oral soft tissue reconstruction is important in restoring function, esthetic, and elevating quality of life after surgical resections of malignant oral cavity lesions. In large defects pediculated regional flaps or free flaps are needed (1).Huge maxillary defects are critical for reconstruction. Palate separates nose from oral cavity. Communications of these cavities after surgical resection of palatal lesions lead to difficulty in speech, nutritional intake, and hygiene maintenance (2).

Reconstruction of maxillary defects that includes alveolar processes and hard palate is difficult. Just periphery of the flap is in contact with bed, therefore, most of the blood supply depends on feeding vessels of the flap Then, and the diffusion from recipient bed has little role. Submental artery island flap is an effective way to solve this problem. Large skin paddle, its axial pattern blood supply, low morbidity of donor site, and vicinity with oral cavity are among advantages of this flap.

## Case Reports

General features of two edentulous patients with large verrucous carcinoma of palate are listed (Table 1).

**Table 1:** General information of two patients with verrocous carcinoma of hard palate.

case	Age/sex	size of lesion	follow up	present state
**1**	72/M	T4 (X >4cm)	3.5 years	no recurrence
**2**	68/M	T4 (X >4cm)	1 year	no recurrence

**Fig 1:**
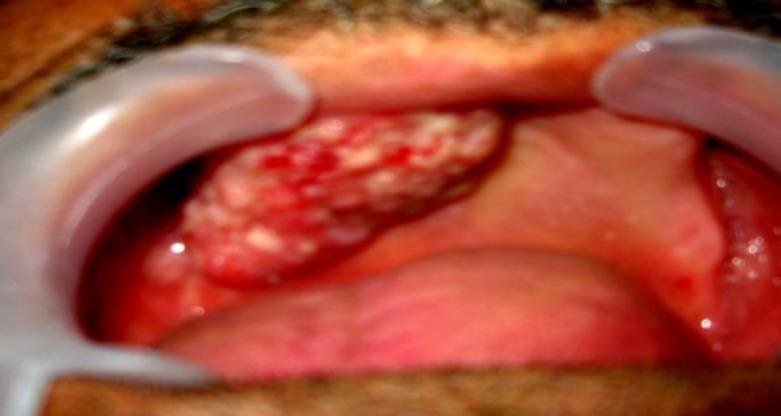
Large exophytic,papillary mass of the alveolar ridge

**Fig 2:**
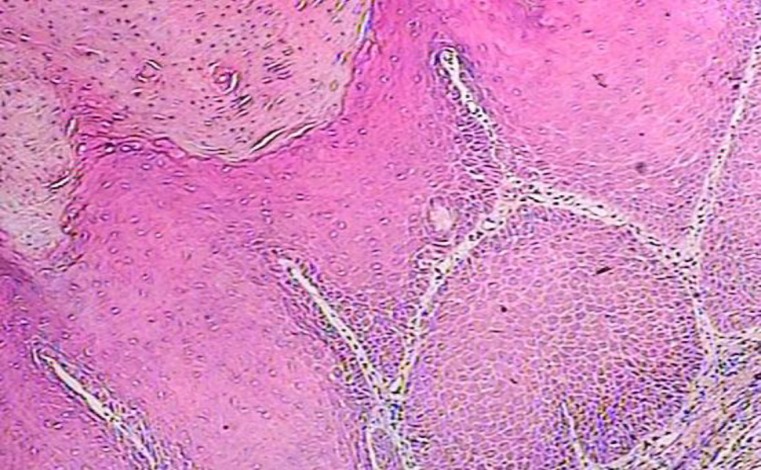
Histopathologic findings show abundant keratin production and a verruciform surface (100× hematoxilin-eosin).

*Case* *1:* A 72-year-old man with a large exophytic, papillary mass of the alveolar ridge referred to the Ghaem hospital (fig.1). Incisional biopsy showed epithelial hyperplasia with a papillary surface and keratin plugging (Fig.2).

**Fig 3:**
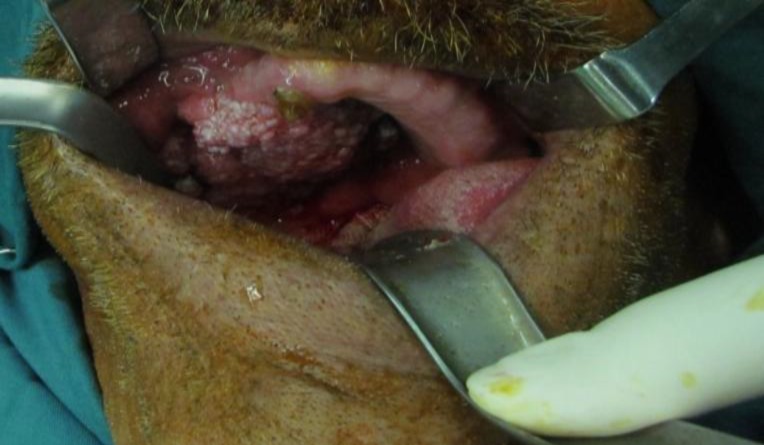
Extensive papillary lesion of the maxillary vestibule and hard palate.

*Case* *2:* The patient had large biopsy proven verrocous carcinoma of hard plate that extended from midline to the right tuberosity. It extended laterally to upper vestibule and medially to midline (Fig.3). It was resected under general anesthesia with 1cm safety margins.

*Surgical method*

**Fig 4:**
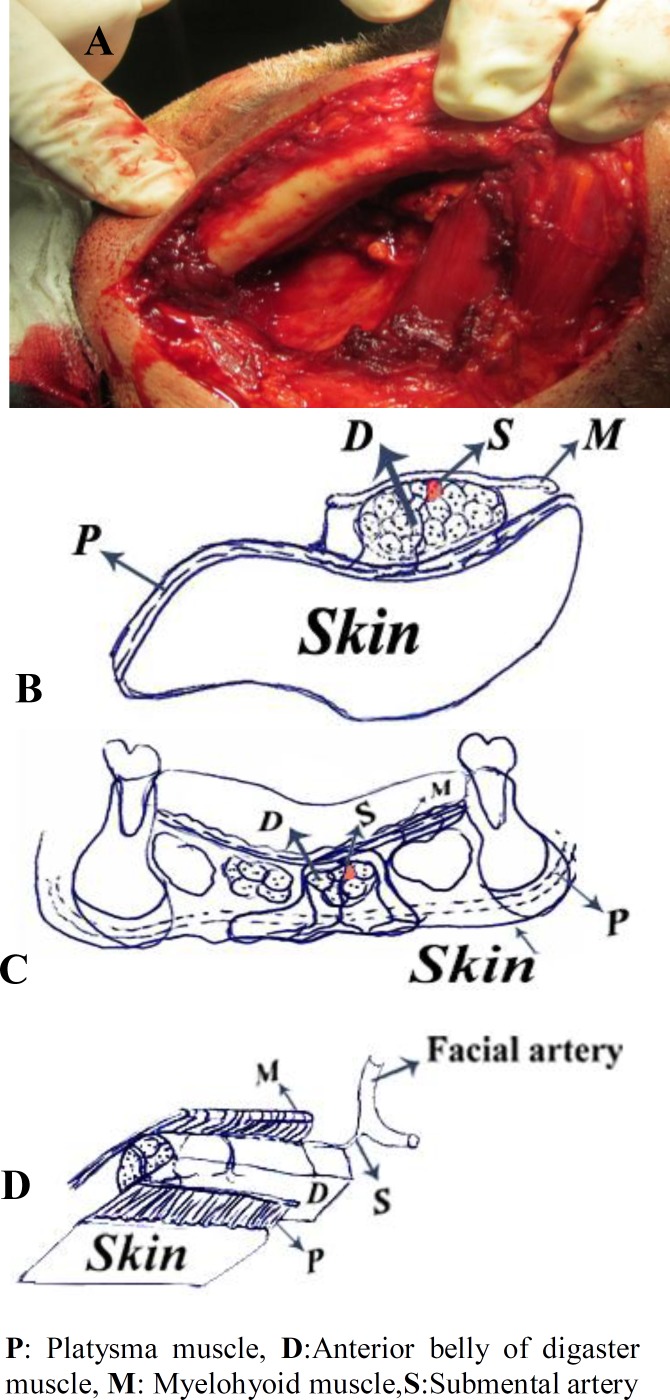
**A. **Myelohyoid (M) and digaster (D) muscle remained undisturbed in nonpedicle part of paddle**. B,C and D** Schematic view of submental flap.

Submental Artery Island flap was used for immediate reconstruction of post maxillectomy defects. Donor site was submental area with 1cm distance from inferior mandibular border. Paddle shape was fusiform. Width of the paddle depends on skin laxity. In elderly patients with excess skin in the neck, we can choose wide skin paddles while yet primary closure of donor site is possible. After designing skin paddle the, first step was to identify facial artery and vein. After that, dissection begins from nonpedicle side and extends toward midline. Dissection was made in subplatysmal plane. In middline, anterior belly of digaster and myelohyoid muscle were included in pedicle side dissection (Fig.4). 

**Fig 5:**
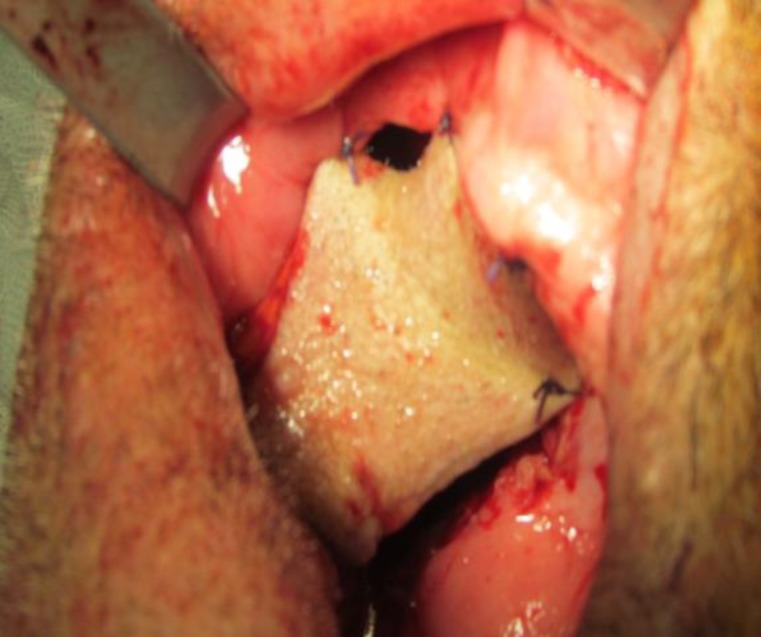
Submental flap brought to hemimaxil- lectomy defect.

Flap pedicle contains submental artery and vein without skeletenizing them. Submucous tunnel was created between vestibular border of post maxillectomy defect and to extraoral incision. Submental flap was brought to the oral cavity via this tunnel and was anchored to the intact remaining hard plate with bone sutures by 2-0 vicryl® suture (Fig.5). 

All skin paddles was brought out of tunnel and sutured to the periphery of maxillectomy defect edge to edges. Donor site closed primarily. Patel modification of Submental flap was used in the reconstruction (9).

## Discussion

Submental artery Island flap can be used as a pedicle or free flap (3). Blood supply of this flap can be retrograded based on facial artery or reverse flow based on angular artery (4,5). Bipedicle submental flap is also available (6). Table 2 shows various modifications of this flap (7-10).

Extensive subdermal plexus between right and left submental arteries makes skin paddle predictable based on one submental artery. Facial artery and vein should be intact, so this flap contraindicate in reconstruction of malignancies with clinical positive neck (N+) or previous neck dissection.

**Table 2:** Submental flap and its modification.

Author/year	Reference	Type of flap	Components of flap in pedicle half	Advantage
**Martin /1993**	7	Fasciocutaneous	S-P	Thin flap
**Gurran /1997**	8	Myocutaneous	S-P-D	Better venous drainage
**Patel /2007**	9	Myocutaneous	S-P-D-M	Better submental artery protection
**Oucik / 1999**	10	Osseomyocutaneous	S-P-D-M-B	Composite flap

S: Skin. P: Platysma. D: Anterior belly of digaster muscle. M: Myelohyoid muscle. B: Bone from inferior mandibular border. Components of flap in non pedicle half includes skin and platysa. (S-p)

In malignant oral lesions that do not need neck dissection like verrocous carcinoma or sarcomas this flap is very useful (11). Supraomohyoid neck dissection with meticulous preservation of facial artery can be accompanied with this flap (12).

**Fig 6:**
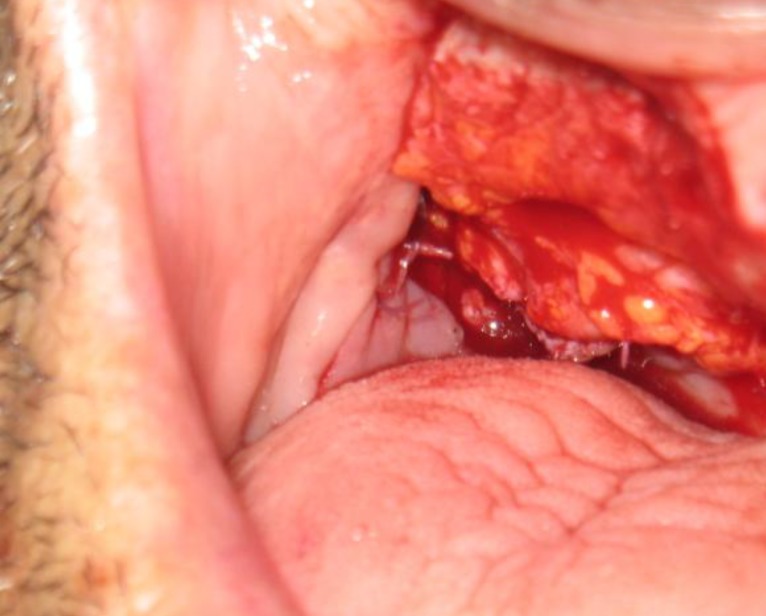
Hair removal 6 weeks later.

Since the hairy nature of this flap is troublesome in intraoral reconstructions in male patients, secondary revision after 6 weeks is required (Fig. 6). Despite this problem, this flap is used in intraoral reconstructions of male patients for its reliability and ultimate soft tissue coverage.

In Uppin research, 14 out of 20 patients were males, in Multinu article 8/12 and in Sebastian article 19/30 Submental Artery Island flaps were used in male patients for orofacial reconstruction (13-15). Vicinity of donor site and hemimaxillectomy defect compensated small pedicle length of the flap. Donor site closed primarily and was hidden in the submental area. In hard palate reconstruction, skin surface of the flap faced towards oral cavity and the raw surface of the flap that contains muscles faced nasal cavity, therefore, small oozing in the night after surgery is anticipated about which the nursing staff members should be informed.

The technique is relatively simple and reproducible. Short length of hospital stay is another advantage of this flap. It has minimal donor site morbidity. Temporary or permanent palsy of marginal mandibular nerve is a possible complication of this flap.

It is important to provide fixation of submental flap to the remaining palate to protect and prevent possible dehiscence of flap interface or total detachment of submental flap from reconstructed palate. This study solve this problem by securing submental flap to remaining palate by bone sutures.

## Conclusion

Submental Artery Island flap is an effective and reliable method for intraoral reconstruction of large soft tissue defects of oral cavity. Donor site morbidity is low and remaining scar is inconspicuous. Head and neck surgeons familiar with facial artery and its branching pattern make this flap an appropriate choice for clinical practice.

## References

[1] Howaldt HP, Bitter K (1989). The myocutaneous platysma flap for the reconstruction of intraoral defects after radical tumour resection. J Craniomaxillofac Surg.

[2] Genden EM, Buchbinder D, Urken ML (2004). The submental island flap for palatal reconstruction: a novel technique. J Oral Maxillofac Surg.

[3] Tang M, Ding M, Almutairi K, Morris SF (2011). Three-dimensional angiography of the submental artery perforator flap. J Plast Reconstr Aesthet Surg.

[4] Chen WL, Li JS, Yang ZH, Huang ZQ, Wang JU, Zhang B (2008 Jun). Two submental island flaps for reconstructing oral and maxillofacial defects following cancer ablation. J Oral Maxillofac Surg.

[5] Carpentier S, Lebeau P, Vandurne B, Gheerardyn R, Vanwijck R, Lengelé B (2008). The versatility of the sub-mental flap: a case report. J Plast Reconstr Aesthet Surg.

[6] Demir Z, Kurtay A, Sahin U, Velidedeoğlu H, Celebioğlu S (2003). Hair-bearing submental artery island flap for reconstruction of mustache and beard. Plast Reconstr Surg.

[7] Martin D, Pascal JF, Baudet J, Mondie JM, Farhat JB, Athoum A (1993). The submental island flap: a new donor site. Anatomy and clinical applications as a free or pedicled flap. Plast Reconstr Surg.

[8] Curran AJ, Neligan P, Gullane PJ (1997). Submental artery island flap. Laryngoscope.

[9] Patel UA, Bayles SW, Hayden RE (2007). The submental flap: A modified technique for resident training. Laryngoscope.

[10] Ducic Y, Hilger PA, Peters MD (1999). The in vitro evaluation of a local pedicled osteomyocutaneous mandibular flap for the reconstruction of composite mandibular defects. J Oral Maxillofac Surg.

[11] Komis C, Lagogiannis GA, Faratzis G, Rapidis AD (2008). Synovial sarcoma of the tongue: report of a case and review of the literature. J Oral Maxillofac Surg.

[12] Varghese BT (2011). Optimal design of a submental artery island flap. J Plast Reconstr Aesthet Surg.

[13] Uppin SB, Ahmad QG, Yadav P, Shetty K (2009). Use of the submental island flap in orofacial reconstruction-a review of 20 cases. J Plast Reconstr Aesthet Surg.

[14] Multinu A, Ferrari S, Bianchi B, Balestreri A, Scozzafava E (2007). The submental island flap in head and neck reconstruction. Int J Oral Maxillofac Surg.

[15] Sebastian P, Thomas S, Varghese BT, Iype EM, Balagopal PG, Mathew PC (2008). The submental island flap for reconstruction of intraoral defects in oral cancer patients. Oral Oncol.

